# Bridging single-molecule and genome-wide studies of cellular mRNA translation

**DOI:** 10.1261/rna.080824.125

**Published:** 2026-04

**Authors:** Adam Koch, Kotaro Tomuro, Taisei Wakigawa, Tatsuya Morisaki, Shintaro Iwasaki, Timothy J. Stasevich

**Affiliations:** 1 Department of Biochemistry and Molecular Biology, Colorado State University, Fort Collins, Colorado 80523, USA; 2 RNA Systems Biochemistry Laboratory, Pioneering Research Institute, RIKEN, Wako, Saitama 351-0198, Japan; 3 Department of Computational Biology and Medical Sciences, Graduate School of Frontier Sciences, The University of Tokyo, Kashiwa, Chiba 277-8561, Japan

**Keywords:** mRNA translation, ribosome profiling, single-molecule imaging, fluorescence microscopy, live-cell imaging

## Abstract

The translation of mRNA is a tightly regulated, energy-intensive process that drives cellular diversity. Understanding its control requires tools that can capture behavior across scales. Over the past two decades, two complementary techniques have emerged that have transformed our understanding of mRNA translation within cells: ribosome profiling (Ribo-seq) and live, single-molecule imaging. Ribo-seq provides genome-wide, codon-level maps of ribosome positions, revealing pause sites, novel open reading frames, and global translation efficiencies. In contrast, live, single-molecule imaging visualizes translation on individual mRNAs in living cells, uncovering heterogeneous initiation, elongation, pausing, and spatial organization in real time. Together, these methods offer complementary strengths—molecular breadth versus temporal and spatial precision—but are rarely applied in tandem. Here, we review their principles, key discoveries, and recent innovations that are bringing them closer together, including endogenous tagging, higher-throughput imaging, absolute calibration, and spatially resolved footprinting. Integrating these approaches promises a unified, multiscale view of translation that connects the dynamics of individual ribosomes to genome-wide patterns of protein synthesis.

## INTRODUCTION

The translation of mRNA into protein by ribosomes is a defining feature of life. The process is tightly regulated and represents one of the largest energy investments a cell makes, highlighting its importance as a key driver of phenotypic diversity in both health and disease. Translation is a dynamic process: initiation, elongation, termination, and ribosome recycling are actively regulated across cell types, environments, and even individual mRNAs, resulting in complex and heterogeneous outputs that help define and tune cellular phenotypes. Deciphering these multiscale dynamics is challenging, so experimental methods are needed to accurately quantify mRNA translation at all levels, from organisms and tissues all the way down to individual molecules.

Over the last 15–20 years, major advances have reshaped how mRNA translation is measured in cells. Ribosome profiling (Ribo‐seq), introduced in 2009 (Ingolia et al. 2009), established a genome-wide, codon-resolved view of ribosome movement along transcripts and remains the benchmark for translation analysis. As a population snapshot, however, it necessarily averages out cell-to-cell and transcript-specific variability. Live-cell, single-molecule imaging of mRNA translation—referred to here as nascent chain tracking (NCT) for short—emerged later as a live-cell approach that visualizes translation along individual mRNA molecules. Although NCT can resolve transcript-specific variability, it has inherently lower throughput than Ribo-seq, making its observations more difficult to generalize (Morisaki et al. 2016; Pichon et al. 2016; Wang et al. 2016a; Wu et al. 2016; Yan et al. 2016).

Together, Ribo-seq and NCT offer complementary perspectives (Table 1). Ribo-seq provides quantitative, genome-wide occupancy and reading-frame maps, while NCT resolves the dynamic behavior of individual transcripts in space and time. Despite their ideal complementarity, the two methods are rarely done together, limiting cross-validation and preventing direct comparisons between single-molecule and transcriptome-wide measurements. Recent reviews from both fields underscore the need for a conceptual and experimental bridge between them (Ingolia et al. 2019; Morisaki et al. 2024).

**TABLE 1.**
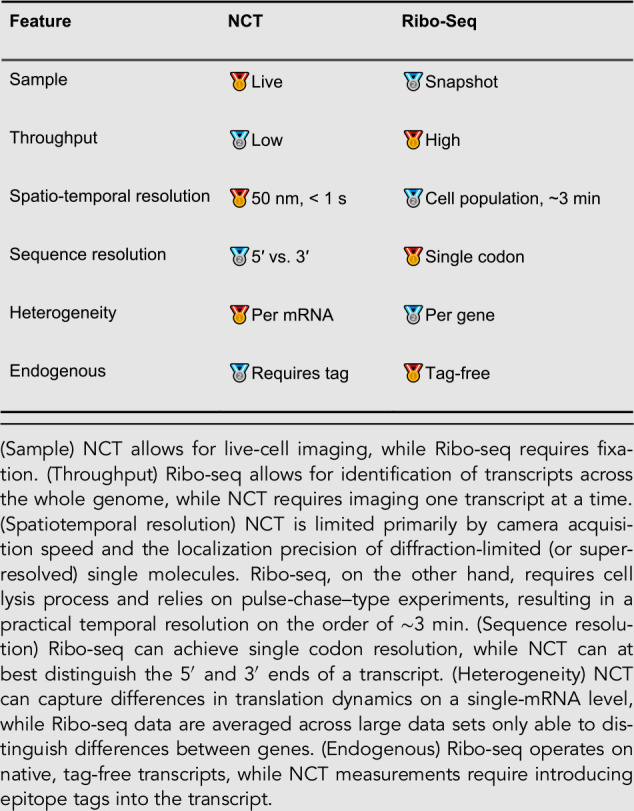
NCT and Ribo-seq are highly complementary techniques

(Sample) NCT allows for live-cell imaging, while Ribo-seq requires fixation. (Throughput) Ribo-seq allows for identification of transcripts across the whole genome, while NCT requires imaging one transcript at a time. (Spatiotemporal resolution) NCT is limited primarily by camera acquisition speed and the localization precision of diffraction-limited (or super-resolved) single molecules. Ribo-seq, on the other hand, requires cell lysis process and relies on pulse-chase–type experiments, resulting in a practical temporal resolution on the order of ∼3 min. (Sequence resolution) Ribo-seq can achieve single codon resolution, while NCT can at best distinguish the 5′ and 3′ ends of a transcript. (Heterogeneity) NCT can capture differences in translation dynamics on a single-mRNA level, while Ribo-seq data are averaged across large data sets only able to distinguish differences between genes. (Endogenous) Ribo-seq operates on native, tag-free transcripts, while NCT measurements require introducing epitope tags into the transcript.

In this review, we outline a roadmap for bridging Ribo-seq and NCT. Our goal is to bring the two communities that use these two powerful technologies closer together. In the sections that follow, we introduce and compare the two technologies and highlight recent innovations that are narrowing the gap between them. We conclude by discussing how the two technologies can be better integrated to provide a more thorough and multiscale understanding of translational regulation. In each section, we begin with the single-molecule viewpoint of NCT and then connect back to the more global view provided by Ribo-seq.

## A TALE OF TWO COMPLEMENTARY TECHNIQUES

To begin, we provide a brief history of NCT and Ribo-seq, pointing out their distinct advantages and disadvantages and highlighting their excellent complementarity (summarized in Table 1).

### NCT core technology

**FIGURE 1.**
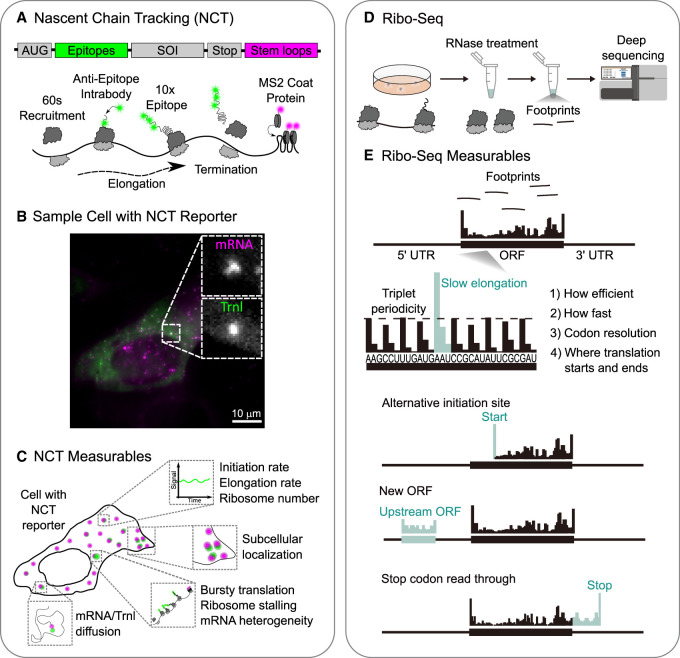
Nascent chain tracking (NCT) and Ribo-seq. (*A*) Schematic of an NCT reporter with an array of epitopes at the N terminus of a sequence of interest (SOI) and an array of stem–loops in the 3′ UTR. The cartoon underneath shows the epitopes being bound by fluorescent anti-epitope intrabodies (green) and the stem–loops being bound by coat proteins (magenta). This amplifies signals, so translation sites can be tracked in living cells. (*B*) Representative image of a living cell expressing the NCT reporter, highlighting punctate mRNA (magenta) and colocalized translation (green). (*C*) Summary of NCT-derived measurables, including single-mRNA/translation-site initiation and elongation rates, ribosome occupancy, subcellular localization, diffusivity, and event-level phenomena such as translation bursts, pausing, and frameshifting. (*D*) Cartoon of the Ribo-seq workflow in which ribosome-protected mRNA fragments are isolated and sequenced to map ribosome positions genome-wide. (*E*) A representative Ribo-seq output showing ribosome footprint density along transcripts, with triplet periodicity and features such as start/stop peaks and pause sites. Ribo-seq-derived measurables include global translation efficiency across the transcriptome, transcript-level ribosome occupancy, codon-specific dwell signatures, pause site mapping, initiation site usage (including upstream ORFs), and readthrough detection.

To track the translation dynamics of individual mRNA molecules in living cells, NCT relies on a two-tag reporter system (Fig. 1). One tag marks the mRNA, and the other tag marks the nascent polypeptide chain as it is still being translated. With this strategy, single translation sites can be pinpointed and their dynamics monitored by tracking spots where signals from both mRNA and nascent polypeptide chains colocalize (Fig. 1B,C; Morisaki et al. 2024).

To tag mRNA, arrays of stem–loops (e.g., 24 × MS2 or 24 × PP7) are encoded in the 3′ untranslated region (UTR) of the reporter (Bertrand et al. 1998; Lim and Peabody 2002; Hocine et al. 2013; Tutucci et al. 2018a). When transcribed, the stem–loops recruit fluorescent coat proteins (e.g., MS2 or PP7 coat proteins [MCP or PCP]) that are coexpressed in cells. These bind tightly and specifically to the stem–loops, which amplifies signals, so individual mRNAs can be tracked for long periods of time (Fig. 1A, magenta). Analogously, to tag the nascent polypeptide chain, arrays of peptide epitopes (e.g., 24 × GCN4 or 10 × HA) are encoded at the N-terminal end of the reporter protein (Morisaki et al. 2024). Soon after being translated, the epitopes recruit fluorescent intrabodies (intracellular antibodies) that are coexpressed in cells. There are many kinds of intrabodies that can be used, including single-chain variable fragments (scFv) (Tanenbaum et al. 2014), fragmented antibodies (Fabs) (Morisaki et al. 2016), or camelid single-chain nanobodies (Muyldermans et al. 2009). These intrabodies bind the epitopes tightly and specifically, which amplifies signals from individual nascent polypeptide chains (Fig. 1A, green). Because translation occurs within polysomes, more than one polypeptide chain is translated at the same time along an individual mRNA, leading to additional signal amplification at individual translation sites.

NCT makes it possible to measure translation site mobility, subcellular localization, and ribosome elongation, initiation, and stalling kinetics (Fig. 1C; Morisaki and Stasevich 2018). Variants of NCT further broaden what can be measured. Tethering cassettes can recruit reporters or effectors to defined locations. Membrane anchoring via CAAX fused to MCP/PCP slows mRNA diffusion and improves imaging conditions for long-term tracking (Khuperkar et al. 2020; Latallo et al. 2023; Livingston et al. 2023; De La Cruz et al. 2025; Galindo et al. 2025), while ER-proximal targeting captures cotranslational recruitment to the ER (Gómez-Puerta et al. 2022; Choi et al. 2026). Complementary stem–loop cassettes, like those from the λN-BoxB system (Coller and Wickens 2007), can also be used to tether regulatory proteins directly to a tagged reporter mRNA and thereby acutely tune initiation and decay (Cialek et al. 2022). To improve imaging signal-to-noise, many reporters also append degrons, such as an ornithine decarboxylase degron (ODC) or auxin-inducible degron (AID), to ensure mature proteins are rapidly cleared while nascent chains remain bright (Lee et al. 2016; Wu et al. 2016).

### NCT key findings

NCT has uncovered broad heterogeneity in translation dynamics among even genetically identical mRNAs. Live measurements in combination with quantitative fitting, often based on a totally asymmetric simple exclusion process (TASEP) (Aguilera et al. 2019; Boersma et al. 2019; Lamberti et al. 2025), place initiation rates at approximately two to five ribosomes/min (with variability observed across transcripts and conditions) and elongation rates between ∼0.5 and 18 codons/sec in human cell lines (Morisaki et al. 2016; Pichon et al. 2016; Wang et al. 2016a; Wu et al. 2016; Yan et al. 2016; Galindo et al. 2025; Lamberti et al. 2025). Even faster elongation rates have been measured in developing embryos, ranging from ∼4 codons/sec to as high as 35 codons/sec (Dufourt et al. 2021; Chen et al. 2024; Pizzey et al. 2025). Recent precise measurements with stopless circular RNAs (socRNAs) have demonstrated that individual ribosomes can translate up to 36,000 codons with high fidelity at rates of ∼2.5 codons/sec (Madern et al. 2024).

By tracking single mRNAs for hours, NCT has further revealed that translation can occur in bursts, with long OFF intervals punctuated by ON periods, the duration and strength of which are modulated by 5′ UTR structure (Wu et al. 2016; Livingston et al. 2023; Blake et al. 2024). Stochastic modeling has been used to extract ON/OFF transition rates and estimate burst sizes (Koch et al. 2020; Livingston et al. 2023; Morisaki et al. 2024). Bursts have also been observed on bicistronic NCT reporters, with IRES-driven bursts typically shorter and rarer than cap-driven bursts, although the situation can reverse under oxidative or ER stress (Koch et al. 2020).

Building on these measurements, NCT has helped pinpoint where ribosomes deviate from uniform elongation. Ribosomes have been observed to slow down, queue, collide, or lose fidelity at problematic sequences. Poly(A)/polybasic tracts (Goldman et al. 2021), structured frameshift stimulatory elements (Lyon et al. 2019), cotranslational targeting pauses (such as XBP1u) (Gómez-Puerta et al. 2022), proline-rich motifs (Watkins et al. 2025), and mRNA stem–loops (Madern et al. 2025) have all been shown to create measurable slowdowns, as recently reviewed in Watkins et al. (2025). Such slowdowns increase the probability of ribosome queues and collisions. Although these are generally considered bad, NCT studies have shown they can have beneficial consequences. The slow clearance of long ribosome queues, for example, provides time for selective targeting by quality control machinery (Goldman et al. 2021), whereas transient ribosome collisions can reduce ribosome pausing on difficult sequences, a process called “ribosome cooperativity” (Madern et al. 2025). Multiframe NCT reporters have also been developed to image and quantify noncanonical translation, including ribosomal frameshifting (Lyon et al. 2019), upstream open reading frames (uORFs), downstream open reading frames (dORFs), stop-codon readthrough (Boersma et al. 2019), and multiframe translation along nucleotide repeat expansions (Latallo et al. 2023).

As NCT is a microscopy-based approach, imaging where translation occurs within cells is straightforward. Translated mRNAs are generally distributed across the cytoplasm (van der Salm et al. 2025), but can be enriched near organelles, such as for ER-targeted transcripts and TOP-mRNA that are regulated by mTOR effectors (Katz et al. 2016; Voigt et al. 2017; Cioni et al. 2019). Within stress granules, mRNAs tend to be translationally silent (Khong et al. 2017; Moon et al. 2019; Helton et al. 2025), although certain stress-induced transcripts can still be translated within stress granules (Mateju et al. 2020). Similarly, mRNAs associated with Ago2/miRNA are translationally repressed and later accumulate in P-bodies, where they tend to remain translationally silent (Cialek et al. 2022).

Finally, NCT has provided evidence for coupling between ribosome elongation and other aspects of translational regulation, such as ribosome initiation and mRNA decay. Elongation through nonoptimal sequences has been shown to dampen initiation by reducing eIF4E/eIF4G engagement (Barrington et al. 2023). These results are consistent with a more recent study that combined imaging and computational modeling (Lamberti et al. 2025). Such dynamic coupling is thought to maintain an even distribution of ribosomes along transcripts, presumably to prevent ribosome traffic jams. Conversely, boosting ribosomal flux has been observed to accelerate mRNA decay, tying instantaneous ribosome traffic to transcript lifetimes (Dave et al. 2023).

### Advantages and disadvantages of NCT

NCT has several advantages: First, NCT provides gold-standard spatiotemporal resolution, with ∼50 nm localization precision and subsecond temporal resolution, enabling high-accuracy quantification of ribosome translation dynamics as they occur in real time (Table 1; Morisaki and Stasevich 2018). Second, NCT is performed in live cells, preserving physiological regulation and allowing translation to be observed in its native context with all regulatory factors present at their proper concentrations. Third, NCT can quantify the translation dynamics of individual mRNAs, revealing transcript-to-transcript variability that is invisible to bulk measurements. Because mRNAs and nascent chains are cotracked, translation can also be linked to subcellular position and regulatory context (e.g., translation in the ER or neurites), and dynamic changes can be monitored through state switches and drug/stress responses (Boersma et al. 2019). With the aid of multiplexed reporters, ratiometric comparisons between different modes of translation on the same transcript are also possible (Boersma et al. 2019; Lyon et al. 2019; Koch et al. 2020).

Despite its strengths, NCT has several key limitations: First, throughput has remained low, with experiments typically analyzing only tens to hundreds of cells and tracking limited to just one or two transcript species at a time. This limits population-wide generalization. Second, NCT relies on engineered reporters with large epitope tags and high-affinity probes. These can interfere with normal protein function, conflict with N-terminal signal peptides, or alter transcript stability and localization (Tutucci et al. 2018b; Li et al. 2022b). Third, a quantifiable signal furthermore requires careful tuning of probe expression, binding site occupancy, and binding kinetics; poor optimization of these parameters can bias analyses toward brighter, more isolated translation sites. Fourth, NCT lacks positional resolution along the mRNA sequence; even if the epitope tag is placed at the N terminus, only elongating ribosomes that have synthesized the tag can be detected, and it remains unclear where those ribosomes are positioned relative to the transcript as a whole.

### Ribo-seq core technology

Ribo-seq provides insights into genome-wide translational dynamics by deep sequencing the RNase-generated short mRNA fragments protected within ribosomes engaged in translation (Ingolia et al. 2009; Tomuro and Iwasaki 2025). In a typical workflow (Fig. 1D), the positions of the ribosomes are stabilized during cell lysis by treating the cells with cycloheximide, after which the cell lysate is digested by nucleases (McGlincy and Ingolia 2017; Mito et al. 2020). The resulting ∼20–30 nt ribosome footprints are purified, converted to libraries, deep-sequenced, and mapped back to the transcriptome to reveal which mRNAs are being translated at what codon positions and with what relative density (Ingolia et al. 2019). In contrast to NCT's single-mRNA trajectories, Ribo-seq offers a genome-wide, codon-resolved view of translation that can be repeated across conditions, cell types, or species to reconstruct entire translational landscapes.

Ribo-seq provides breadth and quantitative depth that complement NCT's single-molecule resolution. In one experiment, Ribo-seq can comprehensively survey which mRNAs are being translated across the entire transcriptome and how efficiently. Because footprints pinpoint the positions of ribosomes, Ribo-seq can map the exact locations of ribosomes that are engaged in translation along each mRNA molecule. This enables the detection of features such as start sites, pause sites, and reading frames (Fig. 1E).

### Ribo-seq key findings

Ribo-seq has revealed global trends in translation dynamics. By coupling the translation initiation inhibitor harringtonine with Ribo-seq, it is possible to estimate the population-average elongation rate by tracking the progressive ribosome-free region along ORFs (Ingolia et al. 2011). This strategy has been employed to quantify elongation rates in both mouse embryonic stem cells and human cells, yielding values of ∼5.6 and ∼4.1 codons/sec, respectively (Ingolia et al. 2011; Tomuro et al. 2024). Furthermore, organ-specific Ribo-seq in mice revealed that translation elongation speeds can vary by over 50% between tissues (e.g., ∼6.8 codons/sec in liver tissue, ∼5.0 in kidney tissue, and ∼4.3 in skeletal muscle tissue) (Gerashchenko et al. 2021). This framework has also been applied to another protein synthesis system in the mitochondria (Richter-Dennerlein et al. 2015; Kummer and Ban 2021). Coupling retapamulin with mitochondrial immunoprecipitation followed by Ribo-seq (MitoIP-Thor-Ribo-seq) revealed that the elongation rates of mitochondrial ribosomes were ∼0.5 and ∼1.0 codons/sec in human and mouse mitochondria, respectively (Wakigawa et al. 2025). This suggests that mitochondrial translation occurs significantly more slowly than cytosolic translation, even within the same cell.

By measuring ribosome occupancy among codons, Ribo-seq has revealed that kinetic alterations in ribosome traversal or ribosome pauses can occur in a wide range of mRNA by multiple mechanisms. Early Ribo-seq studies found that the elongation rate was uniform and not obviously correlated with codon rarity (Ingolia et al. 2011; Li et al. 2012), challenging the classical assumption that rare codons slow down translation (Sørensen and Pedersen 1991). However, more sensitive Ribo-seq studies have since shown that synonymous codon choice and codon optimality positively affect elongation kinetics (Gardin et al. 2014; Hussmann et al. 2015; Weinberg et al. 2016; Mohammad et al. 2019; Wu et al. 2019) at least in yeast and *Escherichia coli*. Beyond codon bias, mRNA secondary structures can impede ribosome movement, due to structured RNA regions or mRNA folding in front of the ribosome that often coincide with footprint pile-ups (Yang et al. 2014; Mizrahi et al. 2018). Chemical modifications on mRNA have also been implicated in altering ribosome transit speeds (Mao et al. 2019; Zhou et al. 2024). Cellular stress conditions that deplete charged tRNAs, such as amino acid depletion, globally slow translation, a phenomenon that Ribo-seq can capture as increased ribosome occupancy at codons decoded by scarce tRNAs (Darnell et al. 2018). Notably, Ribo-seq has highlighted a role for the nascent polypeptide itself in modulating elongation. Runs of positively charged amino acids interacting with the ribosomal exit tunnel can stall translation, as can certain motifs like proline-rich sequences (Charneski and Hurst 2013; Schuller et al. 2017; Arpat et al. 2020; Han et al. 2020; Zhao et al. 2021; Brischigliaro et al. 2024). These diverse insights, ranging from codon optimality to the effects of nascent peptides, have largely emerged from advanced Ribo-seq experiments, providing a comprehensive overview of how sequence and context influence translation elongation across the transcriptome.

When a ribosome pauses for an unusually long time, trailing ribosomes can catch up and collide, forming a stacked complex. Ribo-seq adaptations that isolate disome footprints (∼60 nt) or even trisome footprints (∼90 nt) have mapped collision “hotspots” transcriptome-wide. Disome profiling or Disome-seq has mapped collision sites transcriptome-wide across systems, including human cells and zebrafish embryos (Han et al. 2020), mouse liver and ES cells (Arpat et al. 2020; Tuck et al. 2020), plants (Kim et al. 2021), yeast (Meydan and Guydosh 2020; Zhao et al. 2021), bacteria (Fujita et al. 2022), and viral contexts (Bhatt et al. 2021). These collisions can lead to the queueing of multiple ribosomes on the mRNA and serve as a trigger for downstream quality control pathways. Specifically, the cell has evolved ribosome-associated quality control (RQC) systems to recognize collided ribosomes and rescue or degrade the aberrant translation complexes (Joazeiro 2019; Sitron and Brandman 2020). Together, these findings suggest that ribosome collisions are a general consequence of translational pausing and that they play a key role in maintaining translational homeostasis by activating stress and quality control responses.

One of the most surprising outcomes of Ribo-seq is the discovery that translation is far more widespread than previously thought. Ribosome footprints are detected not only on canonical ORFs of mRNAs but also in untranslated regions and even supposedly noncanonical ORFs (ncORFs) (Couso and Patraquim 2017; Limbu et al. 2024; Tong and Martinez 2025). Ribo-seq derivatives assisted with harringtonine or lactimidomycin, which freeze ribosomes at the start codon but not elongating ribosomes (Ingolia et al. 2011; Lee et al. 2012), allow a more sensitive survey of ncORFs de novo. Moreover, diverse ncORF detection algorithms were developed and indeed identified tens of thousands of actively translated uORFs, as well as small ORFs (smORFs) embedded in long noncoding RNAs. These translation events are collectively expanding what we consider the “translatome.” Among them, uORFs have emerged as a particularly important class: uORFs in the 5′ UTR can act as regulatory elements that conditionally control translation of the main ORF. Mechanistically, uORFs can repress downstream translation by siphoning ribosomes, which causes delayed or reduced reinitiation at the main start codon by triggering ribosome stalling and premature termination, or by altering mRNA stability in response to cellular signals (Dever et al. 2023). Classic examples include stress-responsive genes like *ATF4*, where translation of uORFs is modulated during the integrated stress response to upregulate the main ORF (Harding et al. 2000; Lu et al. 2004; Vattem and Wek 2004). Ribo-seq has helped catalog such uORFs and even identify which are likely to have functional impacts based on their conservation and peptide output (Andreev et al. 2015; Sidrauski et al. 2015). In addition to the function of the translational control module, recent integrative analyses further support the possibility of uORF for synthesizing functional peptides (Fields et al. 2015; Ji et al. 2015; Calviello et al. 2016; Raj et al. 2016; Xiao et al. 2018; Xu et al. 2018; Clauwaert et al. 2019; Calviello et al. 2020; Chen et al. 2020; Choudhary et al. 2020; Mudge et al. 2022). smORFs in long noncoding RNAs should provide a source of biologically active microproteins. This new paradigm led to the proposal of the term “translon,” which is short for translated region. Similar in use to exon or intron, it is meant to encompass all sequences that are decoded by the ribosome, whether conventional ORFs or not (Świrski et al. 2025).

### Ribo-seq advantages and disadvantages

The ability to detect the positions of ribosomes across the entire transcriptome has made Ribo-seq a powerful discovery tool (Table 1). For example, Ribo-seq readily finds novel ORFs (as discussed above) and can monitor translational responses without prior knowledge of which genes might be involved. Second, Ribo-seq is highly generalizable; it can be applied to virtually any organism or tissue from which high-quality ribosome-protected RNA fragments can be isolated. This allows for unbiased profiling of translation in diverse biological contexts, from bacteria and yeast to human cells and from cultured lines to primary tissues. It can also be used in more complex contexts, such as meta-profiling of microbiomes (Fremin et al. 2020). Third, Ribo-seq yields quantitative data that can be integrated with other genomics. By combining ribosome footprint counts with mRNA abundance from RNA-seq, one can estimate translation efficiency per transcript, infer translational regulation, and identify outlier mRNAs under specific conditions.

Ribo-seq also has significant limitations. First, it is an inherently destructive end-point assay. Cells or tissues must be lysed, meaning that only static “snapshots” of translation can be obtained. Unlike live NCT movies, Ribo-seq cannot capture temporal dynamics directly, such as translational bursts or rapid switching on single mRNAs. Any kinetic or cell-to-cell heterogeneity must therefore be inferred rather than observed directly. Second, Ribo-seq data represent an average over millions of cells and mRNA molecules. While this provides valuable population-level information, fine-grained variability is mostly lost. Although NCT reveals transcript-specific spatial regulation, standard Ribo-seq cannot distinguish between isoforms or transcript variants, as short footprints may map to multiple transcript isoforms. Although strategies such as the use of unique isoform regions or coupling Ribo-seq with long-read sequencing have been developed, separating reads based solely on the abundance of each isoform remains difficult (Wang et al. 2016b; Reixachs-Solé et al. 2020). Third, Ribo-seq protocols are technically challenging and often resource-intensive. They typically require a large amount of input material to obtain sufficient footprints, involve multiple biochemical steps, and necessitate deep sequencing to ensure statistical significance. The subsequent data analysis is complex, involving the alignment of numerous short reads and the careful filtering of noise. Additionally, stabilizing ribosomes for Ribo-seq can alter the results. For instance, pretreating yeast cells with cycloheximide, a common practice, has been found to sometimes create artifactual ribosome pile-ups at specific codons (Hussmann et al. 2015; Duncan and Mata 2017; Santos et al. 2019). Although this is not the case for mammalian tissue culture (Sharma et al. 2021), careful drug use, including postlysis cycloheximide treatment (Ingolia et al. 2012; McGlincy and Ingolia 2017; Mito et al. 2020), should be considered. Finally, Ribo-seq lacks the spatial context that NCT provides. It cannot determine whether a given translation event occurred in the cytosol, near the ER membrane, or within a neuronal process—information that NCT can capture through imaging.

In summary, Ribo-seq and NCT offer highly complementary advantages. Ribo-seq is unparalleled for the unbiased, global profiling of translation and for quantifying the molecular basis of translational control across the genome. In contrast, NCT focuses on individual mRNA to reveal dynamic behaviors and spatial regulation that ensemble methods average out.

## BRIDGING THE GAP BETWEEN NCT AND RIBO-SEQ

**FIGURE 2.**
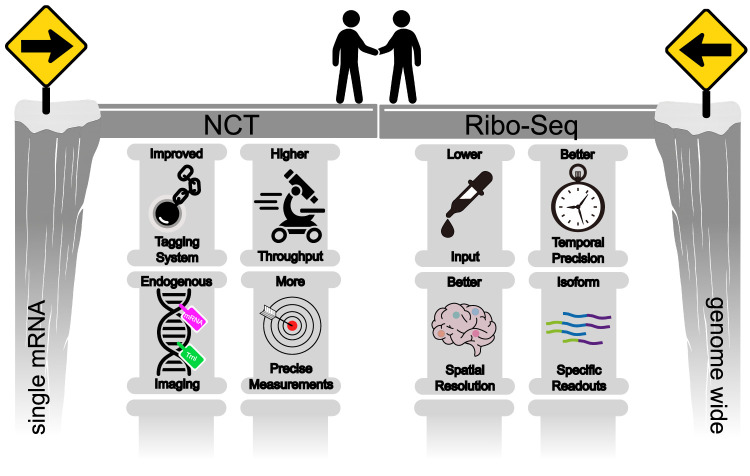
Bridging NCT and Ribo-seq. To bring the two complementary techniques closer together, innovations are required. For NCT (*left*), this requires less invasive tagging strategies, the ability to tag endogenous genes, higher-throughput imaging, and more precise measurements of translation dynamics. For Ribo-seq (*right*), this requires decreasing input requirements, enhancing spatial and temporal resolution, and providing isoform-specific readouts.

Given the very different lengths and timescales at which NCT and Ribo-seq operate, direct comparisons between the two have been challenging. This gap has largely prevented the techniques from being applied side by side. In this section, we highlight recent innovations that are helping to bridge this divide (Fig. 2). On the NCT side, advances in tagging strategies, endogenous imaging, throughput, and precision are converging to enable visualization of more genes with minimal perturbation (Fig. 2, left). On the Ribo-seq side, improvements in input requirements, spatial and temporal resolution, and isoform-specific readouts are enhancing molecular precision (Fig. 2, right). Together, these developments are bringing the two approaches closer, paving the way toward an integrated view of translational regulation.

### NCT innovations that are bridging the gap

#### Improved tagging systems

Unlike Ribo-seq, NCT requires exogenous tags that, when fully saturated with probes, can assemble into very large complexes. For example, a 24× epitope array bound by scFv (∼30 kDa each) yields a complex of roughly 600 kDa per translating ribosome, and when combined with RNA-binding proteins that label the mRNA itself, the total mass can approach the megadalton scale. These large assemblies risk perturbing translation dynamics, motivating efforts to miniaturize and refine the tagging strategy. One approach has focused on using smaller probes: the MoonTag (Boersma et al. 2019) and ALFA-tag (Götzke et al. 2019) use nanobody-based binders, reducing probe size to roughly half that of an scFv (∼15 vs. ∼30 kDa). Complementary advances have targeted the RNA component. The use of improved versions of MS2 stem–loops that do not interfere with mRNA decay (Tutucci et al. 2018b) or translation termination (Li et al. 2022b) permits long-term tracking of individual mRNA molecules with minimal perturbation and high signal-to-noise. More recently, strategies have been developed to selectively degrade unbound MCP, reducing background and increasing contrast (Kuffner et al. 2026; Pham et al. 2026). Finally, bright and photostable fluorophores such as mStayGold (Hirano et al. 2022; Zhang et al. 2024)—which has been used to label both mRNA (Bellec et al. 2024) and nascent peptide chains (Galindo et al. 2025)—enable long-term imaging at lower laser powers, minimizing photobleaching and phototoxicity.

Continued innovation in tagging systems will be needed to further minimize perturbation and more closely approximate the native translation landscape measured by tag-free Ribo-seq. Advances in probe design, including antigen-stabilized fluorophores for background-free imaging (Barykina et al. 2024), reversible Halo ligands that allow mid-experiment dye exchange (Holtmannspötter et al. 2023), and next-generation SNAP-tag variants with improved brightness and kinetics (Kühn et al. 2025), all have the potential to further boost signal-to-noise and enable longer-term tracking of translation in the same cells. At the same time, de novo protein design approaches (Koh et al. 2025) such as RFdiffusion (Watson et al. 2023) and ProteinMPNN (Dauparas et al. 2022) are now being used to generate compact, hyperstable binders with nanomolar-to-picomolar affinities, opening the door to truly minimal peptide tags that are ideally suited for live-cell imaging. Together, these developments point toward a future in which NCT becomes both less invasive and more versatile, enabling simultaneous, quantitative imaging of multiple translation events in living cells with minimal disruption of native physiology.

#### Tracking endogenous genes

Another way to ensure that tagging does not perturb translation dynamics is to integrate the tags directly into an endogenous locus. If the edited cells behave normally and the tagged proteins maintain their proper localization and function, this provides strong evidence that the tagging system is minimally disruptive and that the gene is operating under near-physiological control. By tagging endogenous loci, NCT can better approximate the physiological conditions measured by Ribo-seq while still providing live-cell spatial and temporal resolution. Endogenous SunTag integrations have enabled direct measurements of elongation rates and ribosome density on native polysomes (Pichon et al. 2016). CRISPR knock-ins inserting both MS2/PP7 loops into UTRs together with N-terminal epitope arrays have made it possible to follow native transcripts in their chromatin and translational regulatory contexts, avoiding artifacts from plasmid overexpression (Wiggan and Stasevich 2024).

Alternatively, visualizing native proteins without tags can be done using intrabodies that bind endogenous epitopes. For example, scFv (Sato et al. 2013; Kimura et al. 2015; Galindo et al. 2026) and nanobodies (Rothbauer et al. 2006; Burgess et al. 2012; Schiavon et al. 2020) have been used to trace endogenous antigens in living cells. To extend this approach for translation imaging, epitopes would have to be naturally repeated for signal amplification. One way to achieve this would be to design intrabodies that target repeat epitopes and structural motifs, for example, the heptad repeat in the RNA Polymerase II C-terminal domain (Stiller and Hall 2002), or repetitive protein secondary structures such as TIM barrels (Romero-Romero et al. 2021) or β-propellers (Chen et al. 2011). This strategy could then be combined with new techniques like smLiveFISH (via CRISPR–Csm) (Xia et al. 2024) to colabel the corresponding endogenous mRNA.

#### Higher-throughput imaging

While Ribo-seq can capture translation across the entire transcriptome in a single experiment, NCT has traditionally been restricted to a gene or two in a handful of cells at a time. One route to improving throughput is building on the improved tagging systems discussed earlier. Orthogonal epitope tags (Boersma et al. 2019; Götzke et al. 2019; Zhao et al. 2019; Liu et al. 2021) now allow multiplexed imaging of multiple translation events, while computational design of probe positions can further differentiate targets even within a single detection channel (Raymond et al. 2023). On the microscopy side, automation has streamlined acquisition by minimizing imaging dead time and automatically identifying cells worth imaging (Hiroshima et al. 2024; McSwiggen et al. 2025). In parallel, improvements in imaging larger fields of view, such as oblique line-scan illumination (Driouchi et al. 2025), may allow translation dynamics to be tracked across many more cells simultaneously without compromising single-molecule sensitivity. Trans-scale imaging platforms such as AMATERAS already capture signals across millions of cells in a single field of view, albeit below single-molecule resolution (Ichimura et al. 2025). These approaches have the potential to significantly increase the number of events captured at any given time, which would increase throughput.

A particularly exciting development is the rise of spatially resolved single-cell translatomics, providing molecular-resolution maps of translation (Zeng et al. 2023; Konrad-Vicario et al. 2025). Although currently limited to fixed cells, extending this approach to live cells would bring unprecedented throughput to NCT. How to achieve this is not clear, but one could imagine a large library of barcoded genes containing NCT reporters transfected into cells and imaged with a wide field of view. Cells could then be fixed and the barcode read out to link single-molecule dynamics to large pools of genes simultaneously.

#### More precise readouts of translation dynamics

To accurately compare NCT measurements to Ribo-seq, high precision is required. Most recently, it has become possible to track individual ribosome trajectories using circular mRNAs without stop codons, making it possible to detect transient ribosome collisions and resolve “ribosome cooperativity” (Madern et al. 2025). Joint imaging–modeling frameworks that fit stochastic initiation and elongation models to single-mRNA trajectories have also improved parameter identifiability and revealed coupling between initiation and elongation (Livingston et al. 2023; Raymond et al. 2023; Lamberti et al. 2025). As well, orthogonal initiation controls, such as iron response elements, have been used to synchronize translation onset and improve signal-to-background ratios (Dave et al. 2023). Finally, the coimaging of cofactors at translation sites has begun to connect observed ribosome kinetics to specific molecular mechanisms in vivo (Watkins et al. 2025).

Building on this, there is a need for better calibration standards and cross-assay benchmarking. Measuring the kinetics of translation in different experimental ways but using the same reporter construct would be a good first step. Although harringtonine-induced ribosome runoff (Pichon et al. 2016; Wang et al. 2016a; Yan et al. 2016), fluorescence recovery after photobleaching (FRAP) (Morisaki et al. 2016; Pichon et al. 2016; Wu et al. 2016), and fluorescence correlation spectroscopy (FCS) (Morisaki et al. 2016; Coulon and Larson 2016) have all been used to estimate elongation (and infer initiation), no study has done all three measurements on a single reporter in the same study. This sort of benchmarking could reveal systematic biases and hopefully would lead to a useful gold standard for calibration across laboratories.

### Ribo-seq innovations that are bridging the gap

#### Lower input requirements

Traditional protocols required large amounts of input material and deep sequencing to achieve good data quality (McGlincy and Ingolia 2017; Mito et al. 2020). This was partly because of the multistep workflow (e.g., repeated gel extractions and column purifications) that leads to step-by-step sample loss. Recent innovations have dramatically increased the sensitivity of Ribo-seq, lowering input requirements by orders of magnitude. One strategy has been to simplify library preparation with a one-pot reaction, eliminating low-efficiency ligation steps (Hornstein et al. 2016; Li et al. 2022a; Xiong et al. 2022; Zhang et al. 2022; Zou et al. 2022; Ferguson et al. 2023). A complementary strategy is to amplify footprints before library preparation. Thor-Ribo-seq embodies this approach, as it harnesses T7 RNA polymerase to perform RNA-dependent RNA amplification of ribosome footprints (Shichino et al. 2025). Because this linear amplification occurs immediately after footprint extraction, it minimizes material loss prior to sequencing.

Single-cell Ribo-seq (scRibo-seq) reduces the amount of input material required in single-pot experiments or microfluidic isotachophoresis (ITP), making it possible to resolve the variability of protein synthesis between cells (VanInsberghe et al. 2021; Ozadam et al. 2023). This ultrasensitive method will facilitate the exploration of rare cell types and minute tissue samples that were previously intractable. As protocols improve further in terms of mRNA footprint recovery and amplification, routine single-cell translatome studies may become a reality. This will enable researchers to chart cell-to-cell variability in protein synthesis and discover specialized translation programs in development or disease. An exciting future direction is to integrate low-input Ribo-seq with single-cell transcriptomics or proteomics to connect translational heterogeneity with other layers of gene regulation.

#### Tracing absolute kinetics of translation

Conventional Ribo-seq provides a snapshot of translation and the relative density of ribosomes on each mRNA. While powerful for comparing relative changes (typically as log_2_ fold changes), standard Ribo-seq does not yield absolute kinetic parameters, such as initiation, elongation rates, or even absolute ribosome numbers on the CDS. Although traditional sucrose density gradient ultracentrifugation and following RNA-seq provide a means to detect the loaded ribosomes on mRNA (Sample et al. 2019; González et al. 2025), the separation of the polysomes is generally limited to eight to ten ribosomes per mRNA. To better compare with NCT, new approaches aim to calibrate Ribo-seq data into absolute units. One major innovation is Ribo-Calibration (Tomuro et al. 2024), which introduces a known stoichiometry of ribosome–mRNA complexes as an external standard. In this method, in vitro assembled ribosomes bound to synthetic mRNAs are spiked into the cell lysate prior to nuclease digestion. Because the exact number of ribosomes on the spike-in mRNA is known, the sequencing read counts can be converted into absolute numbers of ribosomes per endogenous transcript. For selected individual mRNAs, the calibrated ribosome numbers were well-aligned to the values obtained with sucrose density gradient ultracentrifugation (Tomuro et al. 2024). This has revealed that the global ribosome load was approximately five ribosomes per 270 nt, with initiation occurring approximately every 22 sec in HEK293 T-REx cells. These values are in excellent agreement with those obtained by NCT in mammalian cells (Morisaki and Stasevich 2018). This analysis was extended to evaluate the translation kinetics of mitochondrial translation (Wakigawa et al. 2025), which suggested much slower dynamics of mitochondrial ribosomes. Similar quantification was also employed to estimate the abundance of disomes. Using synthetic, footprint-sized oligos for spike-in normalization, the calibrated disome-to-monosome ratio in mouse livers was measured, revealing that ∼10% of ribosomes involved in translation reside in collided disomes (Arpat et al. 2020).

Calibrated Ribo-seq has opened the door to measuring kinetics on a transcriptome-wide scale (Tomuro et al. 2024). As well as providing information on average initiation frequencies and elongation speeds, it enables the estimation of ribosome runoff times and the number of protein molecules produced per mRNA before it is degraded (∼1800 times on average). However, current calculated values still rely on the global average elongation rate, which is based on harringtonine chase sampling at a few time points and restricted to mRNAs possessing enough long CDSs (Ingolia et al. 2011). Further refinement of Ribo-seq is required to achieve temporal precision comparable to live imaging. One possible approach is to increase the number of very short chase time points in runoff experiments and also the sequencing depth, which would enable the elongation rate of each mRNA to be determined individually. Overcoming this limitation allows for better mathematical modeling of the ribosome flow on CDS by TASEP (Dao Duc and Song 2018; Riba et al. 2019; Erdmann-Pham et al. 2020; Szavits-Nossan and Ciandrini 2020), further fostering the connection between Ribo-seq and live imaging in the theoretical point of view.

Another critical advance for closing the gap with NCT is developing ways to track translation in the same cell over time, rather than in matched but separate cell populations. Live-cell imaging naturally follows a single cell's translation activity longitudinally, whereas standard Ribo-seq destructively lyses cells at one time point. An intriguing solution is to borrow from minimally invasive transcriptomics. For instance, Live-seq demonstrated that it is possible to profile a living cell and then revisit it later to observe outcomes (Chen et al. 2022). Live-seq uses a tiny hollow nanopipette to extract a small portion of cytoplasmic mRNA from a cell without killing it. The cell remains viable, allowing one to sequence its mRNAs and then let it continue growing or respond to treatments, and possibly sequence it again. Alternative ways for time-resolved transcriptomics through the engineered virus-like particles are also reported (Najia et al. 2024). While those experiments demonstrated their capacity only for transcriptomes, a similar principle could be applied to the translatome.

#### Tracing local translation

The importance of local translation in many systems, ranging from asymmetric egg development to neuronal synapses, is well recognized (Das et al. 2021). For example, neurons localize thousands of mRNAs to distal processes, and local protein synthesis in dendrites and axons is crucial for synaptic plasticity and axon guidance (Cioni et al. 2018; Holt et al. 2019). Manual dissection and fractionation, such as the separation of neuropil fractions, have demonstrated the ability to capture localized translation using Ribo-seq (Biever et al. 2020; Glock et al. 2021). While these approaches confirmed that mRNA localization leads to functionally distinct translation sites, their spatial resolution is limited, and they are not easily generalizable to small organelles or nonneuronal contexts. To achieve finer spatial resolution, researchers turned to proximity labeling strategies that tag ribosomes only in specific subcellular locations. A landmark methodology is BirA–AviTag-mediated proximity-specific ribosome profiling, first introduced by the Weissman lab (Jan et al. 2014; Williams et al. 2014). This approach involves engineering cells to coexpress two components: (1) a spatially localized biotin ligase (*E. coli* BirA) and (2) a mutant ribosomal protein bearing a short AviTag sequence. BirA biotinylates any AviTag-conjugated ribosome within a few nanometers, and selective purification using streptavidin enables decoding of site-specific translation. In the first two papers (Jan et al. 2014; Williams et al. 2014), proximity-specific ribosome profiling was applied to the ER membrane (ERM) in human and yeast cells and outer mitochondrial membrane (OMM) in yeast. A subsequent paper also revealed peroxisomal local translation in yeast (Dahan et al. 2022). Proximity-specific Ribo-seq thus makes it possible to biochemically isolate translating ribosomes from a defined microenvironment within the cell. However, the original method had practical limitations: It required cells to be grown in biotin-depleted conditions to minimize background biotinylation and provided only limited temporal resolution. Additionally, optimizing appropriate locations of BirA/AviTag engineering in each organelle can be laborious.

Recent advances have overcome these issues by adding optogenetic control or using faster labeling enzymes. One solution is to make biotin ligase activity light-inducible, thereby eliminating the need for biotin starvation and enabling precise timing of labeling. For instance, the optogenetic proximity labeling method termed AviTag-specific location-restricted illumination-enhanced biotinylation (ALIBi) has been reported (Zhang et al. 2025). In this system, the BirA enzyme is split into two inactive fragments that are brought together by a light-inducible dimerization domain using the eMag system (Benedetti et al. 2020), effectively creating a BirA that is inactive in the dark and activated by light. In parallel, an optogenetic ribosome tagging tool called LOV-domain-Controlled Ligase for Translation Localization (LOCL-TL) has been developed (Luo et al. 2025). The authors fused a LOV photoactivatable domain (Lee et al. 2023) to BirA such that blue light triggers BirA to biotinylate nearby ribosomes only upon illumination. Both ALIBi and LOCL-TL represent a new generation of optogenetic, spatiotemporally controllable Ribo-seq techniques. In addition to biotin ligases, researchers have employed engineered peroxidases and proximity labeling enzymes to map local translation. APEX2 (Lam et al. 2015) and TurboID (Branon et al. 2018) are enzymes that create highly reactive radicals to tag nearby biomolecules. These have mostly been used to catalog proteomes of organelles, but recently they have been adapted for translatome mapping. In TurboID-mediated Ribo-seq, the idea is to selectively label ribosomes or nascent peptides in a compartment, then isolate them cross-linked with RNA for sequencing (Hacisuleyman et al. 2024). They tethered TurboID to the dendrite, so that they could map the dendritic translatome upon depolarization. A parallel effort is APEX-Ribo-seq (Tomuro et al. 2025). Ribosomes and mRNAs in the vicinity become biotinylated upon hydrogen peroxide addition, and a comprehensive characterization of local translation in 14 distinct organelles has been provided using this method.

The next step is to extend these subcellular spatial Ribo-seq technologies in vivo and to a wider array of organisms and tissues. Optogenetic BirA systems like ALIBi and LOCL-TL could, in principle, be used in living tissues. Similarly, chemical proximity labeling could be deployed in living animals: for example, Halo-seq (Engel et al. 2022) is a recently developed RNA proximity labeling method that uses a small molecule photosensitizer to label RNAs near a protein of interest. The derivative oxidation-induced nucleotide conversion (ONIC)–seq (Lo et al. 2025) harnesses oxidation of guanine (G) to 8-oxoguanine (8-oxoG) in RNAs and detects G-to-cytosine (C) and G-to-thymidine (T) conversion induced during reverse transcription. Microenvironment mapping (μMap) (Knutson et al. 2025) uses photocatalysts to create reactive tags that label biomolecules in a defined microenvironment. Implementation of these methodologies into Ribo-seq should pave the way for monitoring local translation in tissues.

#### Heterogeneous translation readout

NCT excels at revealing variability at the level of individual cells and molecules, making it possible to observe the translation of individual transcripts in real time and resolve heterogeneity that bulk methods might obscure. Due to the nature of Ribo-seq, which requires RNase digestion, distinguishing between isoform-specific translations is difficult. Generally, polysome profiling followed by RNA-seq (transcript isoforms in polysome sequencing or TrIP-seq) (Floor and Doudna 2016; Wang et al. 2016c), subcellular fractionation (Frac)–seq (Sterne-Weiler et al. 2013; Ritter et al. 2024), and translating ribosomal affinity purification (TRAP)–seq (Heiman et al. 2014; Nguyen et al. 2016; Chen et al. 2017; Sapkota et al. 2019; Lyons et al. 2020) could be harnessed for dissecting isoform-specific translation. However, those methods lose the information on the location of ribosomes on mRNA. One key innovation that improves this is Ribo-STAMP (surveying targets by APOBEC-mediated profiling) (Brannan et al. 2021), a technique that marks RNAs that are being translated by leveraging a nucleotide-editing enzyme. The principle is to fuse ribosomes with APOBEC1, which is a cytosine deaminase that catalyzes RNA C-to-uracil (U) conversion on single-stranded RNA substrates (Navaratnam et al. 1993). When the fusion with ribosomal protein RPS2 (uS5) or RPS3 (uS3) is expressed in cells, any mRNA that is actively engaged with a ribosome will be subject to APOBEC1-catalyzed C-to-U editing at sites proximal to the ribosome. This allows detection of translated mRNAs without needing to isolate ribosomes or do footprinting. By combining this approach with long-read sequencing, it was shown that this could yield information on which splice isoforms of a gene are being actively translated in the cell (Jagannatha et al. 2024).

Looking ahead, the principle of using targeted RNA editing to mark actively translated transcripts is expanding the isoform-specific translatome toolkit. Recently, similar editing-based platforms have further broadened these capabilities. For example, modification added to RBP interacting transcript (MAPIT)–seq employs an antibody-guided strategy to recruit RNA editors to transcripts bound by a target protein (Cheng et al. 2025). In MAPIT-seq, a fusion of protein A/G with two deaminases, APOBEC1 and an ADAR2 domain, is used, enabling in situ C-to-U and A-to-inosine (I) editing on RNAs near the protein of interest. This will allow isoform-specific detection of RBP-bound or ribosome-bound mRNAs directly in fixed cells and even tissue sections. Enabling RNA editing in vitro after cell/tissue lysis could be an alternative option for the versatile use of these technologies in the future.

## OUTLOOK

Throughout this review, we have highlighted the opportunities for bridging the gap between NCT and Ribo-seq, as well as the strategies and innovations that are beginning to make this integration possible. These two methods are highly complementary, and the time is ripe to apply them side by side within the same experimental systems. Until now, the NCT and Ribo-seq communities have largely operated independently, with minimal overlap in practice. To our knowledge, only one study to date has applied both methods to the same system (Barrington et al. 2023). A key goal of this review is therefore to raise awareness across both communities and to encourage broader adoption of such combined approaches.

**FIGURE 3.**
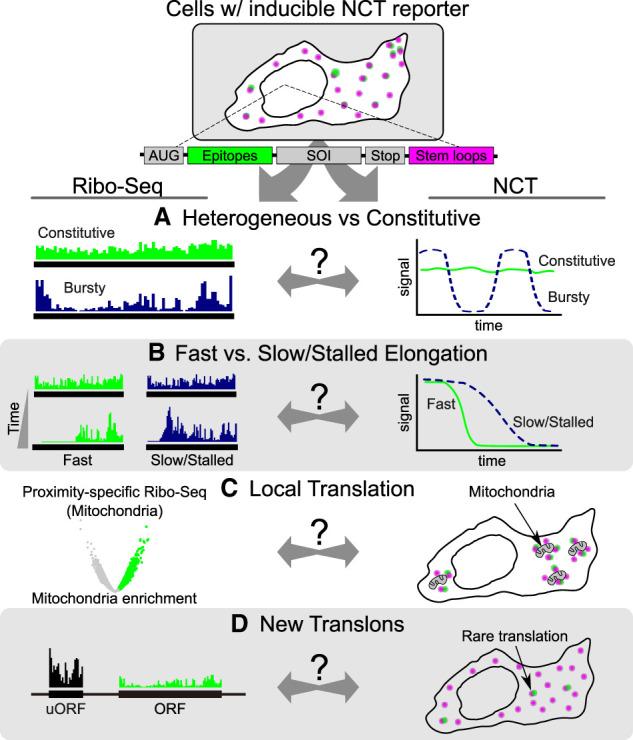
Combining Ribo-seq and NCT. Combined Ribo-seq and NCT studies that use cells that express NCT reporters will help clarify data interpretation. Hypothetical results are shown from four possible experiments with cells expressing NCT reporter transcripts that (*A*) are heterogeneously versus constitutively expressed, (*B*) contain sequences that run off fast or slow in response to initiation inhibitors, (*C*) are locally translated, and (*D*) contain new translons.

As a first step, both methods could be tested for bias; for example, do the large NCT tags inadvertently slow translation dynamics, or do any of the Ribo-seq processing steps unintentionally alter ribosomal occupancies? We can get at these questions by doing the two techniques side by side. By performing Ribo-seq in a cell line that stably expresses NCT tags and probes, for example, we can see if those tags and probes alter ribosomal footprints (Fig. 3). Analogously, by performing NCT based on the Ribo-seq results, we can see if any of the wet/dry processing steps impact NCT translation signals. In this way, each method can serve as a check on the other, building mutual confidence in their complementary readouts.

Second, we should begin to design studies where both techniques are applied to the same experimental system under near identical conditions (Fig. 3). Ribosome runoff experiments, for example, could be used to compare elongation curves directly, helping to establish whether the dynamics captured by NCT align with the temporal snapshots derived from Ribo-seq (Fig. 3B). Developing the ability to predict one type of data set from the other would be a significant advance, making it possible to translate between single-molecule imaging and genome-wide profiling.

To push both methods forward, it will be especially informative to examine the extremes of translation. For instance, what are the upper limits of ribosome initiation and elongation rates? Ribo-seq can identify transcripts with unusually high ribosome density or rapid elongation, which can then be tested directly with NCT to see what these extremes look like in living cells. Conversely, strongly heterogeneous or bursty translation behaviors detected by NCT can be cross-checked with Ribo-seq to see if there are hidden signatures of the variability within the distributions of ribosome footprints (Fig. 3A). These reciprocal comparisons are needed to bridge population-level measurements with single-molecule dynamics.

As the two methods begin to converge on consistent measurements, their integration will offer a more comprehensive view of translational regulation. Combining live-cell NCT data with genome-wide Ribo-seq maps can reveal how translation is organized, coordinated, and controlled across biological contexts, ultimately providing a unified framework that connects molecular dynamics with global patterns of protein synthesis.

## COMPETING INTEREST STATEMENT

S.I. is a member of the editorial board of *Scientific Reports*. The remaining authors declare that they have no competing interests.
